# Supplementary material for: A high-resolution mRNA expression time course of embryonic development in zebrafish
Source: eLife. 2017 Nov 16;6:e30860. doi: 10.7554/eLife.30860 (PMC5690287; doi:10.7554/eLife.30860)
Supplement: Supplementary file 6. [file elife-30860-supp6.zip › biolayout-clusters-files/Cluster090-genes.html]

Cluster090


# Cluster090: Genes

| Ensembl ID | Gene Name | Chr | Start | End | Biotype |
| --- | --- | --- | --- | --- | --- |
| ENSDARG00000008772 | cacng1a | 6 | 19411421 | 19432458 | protein\_coding |
| ENSDARG00000095675 | ccdc141 | 9 | 43272744 | 43340849 | protein\_coding |
| ENSDARG00000019342 | chrnd | 24 | 25538120 | 25546069 | protein\_coding |
| ENSDARG00000103533 | clec14a | 17 | 10086132 | 10087854 | protein\_coding |
| ENSDARG00000032976 | cmlc1 | 5 | 63486421 | 63487216 | protein\_coding |
| ENSDARG00000043395 | crip3 | 17 | 21944636 | 21962999 | protein\_coding |
| ENSDARG00000009839 | fam117bb | 9 | 13149216 | 13241587 | protein\_coding |
| ENSDARG00000042368 | kif26aa | 20 | 27529688 | 27653524 | protein\_coding |
| ENSDARG00000006757 | klhl41b | 6 | 3519734 | 3529383 | protein\_coding |
| ENSDARG00000029764 | mef2ca | 10 | 43716434 | 43826987 | protein\_coding |
| ENSDARG00000074829 | rasip1 | 16 | 12626633 | 12669820 | protein\_coding |
| ENSDARG00000103900 | sept9b | 6 | 22464552 | 22552420 | protein\_coding |
| ENSDARG00000091009 | si:ch211-28p3.4 | 12 | 30448648 | 30452809 | protein\_coding |
| ENSDARG00000037748 | slc43a1b | 1 | 43890189 | 43936755 | protein\_coding |
